# Analysis of the Site-Specific Myoglobin Modifications in the Melibiose-Derived Novel Advanced Glycation End-Product

**DOI:** 10.3390/ijms232113036

**Published:** 2022-10-27

**Authors:** Kinga Gostomska-Pampuch, Jacek R. Wiśniewski, Karol Sowiński, Wieslaw I. Gruszecki, Andrzej Gamian, Magdalena Staniszewska

**Affiliations:** 1 Department of Biochemistry and Immunochemistry, Wroclaw Medical University, 50-368 Wroclaw, Poland; 2 Department of Proteomics and Signal Transduction, Max Planck Institute of Biochemistry, 82152 Martinsried, Germany; 3 Department of Biophysics, Institute of Physics, Maria Curie-Sklodowska University, 20-031 Lublin, Poland; 4 Laboratory of Medical Microbiology, Hirszfeld Institute of Immunology and Experimental Therapy, Polish Academy of Sciences, 53-114 Wroclaw, Poland; 5 Faculty of Medicine, The John Paul II Catholic University of Lublin, 20-708 Lublin, Poland

**Keywords:** mass spectrometry (MS), post-translational modification (PTM), glycation, melibiose

## Abstract

MAGE (melibiose-derived advanced glycation end-product) is the glycation product generated in the reaction of a model protein with melibiose. The in vivo analog accumulates in several tissues; however, its origin still needs explanation. In vitro MAGE is efficiently generated under dry conditions in contrast to the reaction carried in an aqueous solvent. Using liquid chromatography coupled with mass spectrometry, we analyzed the physicochemical properties and structures of myoglobin glycated with melibiose under different conditions. The targeted peptide analysis identified structurally different AGEs, including crosslinking and non-crosslinking modifications associated with lysine, arginine, and histidine residues. Glycation in a dry state was more efficient in the formation of structures containing an intact melibiose moiety (21.9%) compared to glycation under aqueous conditions (15.6%). The difference was reflected in characteristic fluorescence that results from protein structural changes and impact on a heme group of the model myoglobin protein. Finally, our results suggest that the formation of in vitro MAGE adduct is initiated by coupling melibiose to a model myoglobin protein. It is confirmed by the identification of intact melibiose moieties. The intermediate glycation product can further rearrange towards more advanced structures, including cross-links. This process can contribute to a pool of AGEs accumulating locally in vivo and affecting tissue biology.

## 1. Introduction

Advanced glycation end-products (AGEs) constitute a very heterogeneous group of compounds with diverse physicochemical and biological properties that accumulate in the body, leading to disturbances in homeostasis. They are formed during the non-enzymatic process initiated by a glycation reaction between a carbonyl group of reducing sugars or other aldehydes with a basic group of proteins, lipids, and nucleic acids [1]. Glycation is exacerbated in metabolic diseases accompanied by oxidative stress, such as diabetes, atherosclerosis, Alzheimer’s disease, and cancer [2]. To date, only a small percentage of AGEs have been structurally characterized and confirmed in vivo, with many of the glycation products, perhaps crucial for certain pathology, yet to be described.

AGEs can affect the structure and change properties of the modified proteins or cause aggregation, resulting in pathological changes [3]. The type of protein and glycation substrate determine a range of the observed effects. Glucose is the most often studied glycation substrate; an increased concentration in the body causes glycation of albumin and hemoglobin, used as the markers in the monitoring of hyperglycemia [4]. However, the reactivity of glucose is quite weak since, in the cyclic semi-acetal form of the sugar molecule, there is no reactive aldehyde group available for condensation with the amino groups of proteins that occurs only in the open-chain form of a glucose molecule [5]. Other carbohydrates, such as fructose, ribose, and dicarbonyl compounds, i.e., methylglyoxal (MGO) and glyoxal (GLX), are much more reactive, generating AGEs with much higher yield [6,7,8]. Reactive dicarbonyls are formed as byproducts of a glycation process and are also produced in a body by lipid oxidation, degradation of glycated proteins, and glycolysis. These compounds cause carbonyl stress, which may contribute to the degradation or modification of carbohydrates, lipids, proteins, and DNA [9]. Among various reactive carbonyl species formed during protein glycation, the concentration of MGO, GLX, and glycolaldehyde (GA) increases significantly in the plasma of people with hyperglycemia [10]. AGEs formed from these carbonyls contribute to increased protein aggregation and the formation of amyloid deposits [11,12].

The AGE structures described in the literature mainly consider protein modifications on the basic amino acids of lysine and arginine. The glycation of a single lysine residue causes formation of Nε-carboxymethyl-lysine (CML) [13,14], Nε-carboxyethyl-lysine (CEL) [15], pyrraline [16], glycolaldehyde pyridine (GAP) [5], fructoselysine (FL) [17], and a few others. The AGEs consisting of two lysine residues within the same molecule are called lysine–lysine cross-links and are present in a crossline [18], glyoxal-lysine dimer (GOLD), and methylglyoxal-lysine dimer (MOLD) [5]. The modification of a single arginine residue is observed within Nω-carboxymethyl-arginine (CMA), Nω-carboxyethyl-arginine (CEA) [19], hydroimidazolone derived from glyoxal (G-H1) [20], and hydroimidazolone derived from methylglyoxal (MG-H1) [21]. The glycation and cross-linking of lysine and arginine residues results in the formation of pentosidine [22] and cross-linked imidazolone derived from 3-deoxyglucosone (DOGDIC) [5].

Recently, our group has demonstrated a novel unconventional AGE antigen accumulating in several tissues of a human and an animal body [23]. Our study showed the structural in vitro obtained analogous adduct (MAGE) forming during glycation on a variety of proteins exposed to melibiose (mel) in anhydrous conditions. The study employing mass spectrometry and immunochemical analysis identified several proteins modified with MAGE [24] in the human blood of healthy, diabetic, and cancer subjects. MAGE presents different antigenic properties in comparison to AGE formed in a water solution from the same substrates. The identified biological properties of MAGE show genotoxicity studied in vitro on human peripheral blood lymphocytes, melanoma, lung cancer, and colorectal cancer cells, while protein-free adducts prevent cells from this effect [25]. It has also been associated with diabetic complications and atherosclerosis [26,27]. It should be noted that melibiose is produced during food fermentation by bacteria of the genus Bifidobacterium [28,29], Lactobacillus, Lactococcus, Leuconostoc [30], and yeast [31], and it is also found in cocoa beans, honey, and processed soybeans [32]. Supplied with food, melibiose is absorbed in the small intestine through para-cell junctions existing between adjacent enterocytes [33]. This may suggest an exogenous source of mel as a substrate for further MAGE production; however, there are currently no data available on possible MAGE formation in the gut and subsequent absorption into the bloodstream.

Previously, the in vitro synthesized protein-free MAGE structure has been elucidated and is shown to exist as isomers in two forms: cyclic and open [23]. As we have also reported, the dry state and aqueous glycation conditions catalyze various AGEs structures on proteins and unique antigenic properties [34]. However, there is still a need for the determination of detailed protein modifications by melibiose and to understand the formation mechanism. Here, we have taken on the first step and characterized the MAGE product formed in vitro on a model protein myoglobin (MB). The extent and the specific amino acid residues modified during the reaction with mel have been identified. Below we present the mass spectrometry results comparing the MB-mel product (MB modified by mel) generated on a protein during glycation under anhydrous conditions with the AGE adducts formed in an aqueous solution from the same substrates. We also define the basic physicochemical properties and structural changes of the obtained AGE products.

## 2. Results

### 2.1. Identification of AGEs Generated on the Model Protein MB

**Figure 1:**
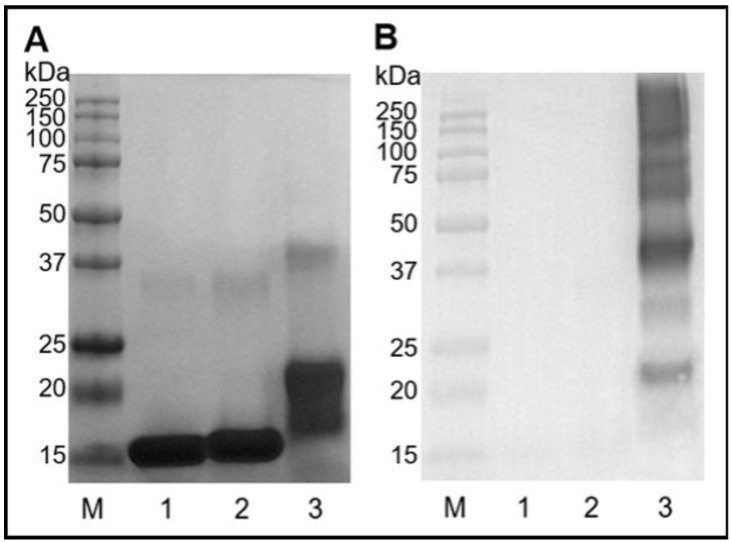
Electrophoretic analysis of MB protein modified by mel in ACG and MWG glycation conditions. (**A**) 12% SDS-PAGE gel stained with Coomassie Blue and (**B**) Western blotting analysis with anti-MAGE antibodies; M—protein marker, 1—native MB, 2—MB-mel ACG, 3—MB-mel MWG.

The model MB protein that we used in the experiments was mainly a monomer migrating in polyacrylamide gel as a molecule of a molecular mass around 16 kDa (Figure 1A, lane 1) and contains a negligible amount of a dimer (32 kDa). In contrast, SDS-PAGE analysis of MB subjected to glycation in the presence of mel revealed the formation of several products depending on a glycation method. MB-mel formed in the conditions of the conventional glycation carried in a solution (ACG) showed two major bands (Figure 1A, lane 2), with the most abundant MB monomer migrating slightly higher than the unmodified MB (Figure 1A, lane 2). There were also products with higher molecular mass, migrating as proteins up to 34 kDa suggesting the presence of MB-dimer with different degrees of mel modification. In contrast, the protein incubated with mel under dry conditions (MWG) showed a spectrum of products being MB monomer with a molecular mass of 18–24 kDa as the major fraction and the MB dimer with different mel loads migrating in the range of 37–45 kDa. This indicates that the MWG conditions favor the generation of the products with a higher degree of cross-linking and heavier mel loads.

The further results of Western blotting analysis (Figure 1B) revealed that an antigen formed from MB and mel in dry conditions (MWG) is distinct from the one synthesized in solution under conventional glycation conditions (ACG). The anti-MAGE monoclonal antibody specifically reacted with the MB-mel MWG antigen (Figure 1B, lane 3). There was no cross-reactivity with the MB-mel ACG or native MB (Figure 1B, lanes 1, 2). This confirmed our previous observation that the MAGE epitope is formed only in dry conditions [23].

### 2.2. Absorption and Fluorescence Properties of MB-mel Products

**Figure 2:**
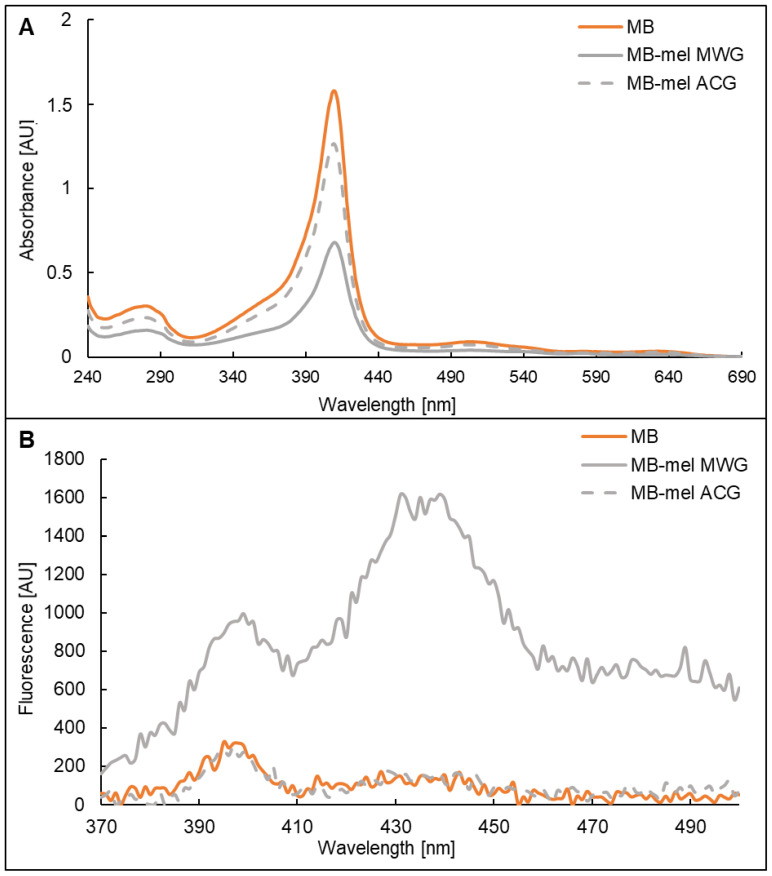
Absorption (**A**) and fluorescence (**B**) spectra properties of the water solutions of MB (orange line), MB-mel MWG (grey solid line), and MB-mel ACG (dashed grey line) at a concentration of 0.5 mg/mL. Absorption spectra were recorded in a range from 240 to 690 nm, and fluorescence spectra upon excitation at 350 nm were recorded in a range from 370 to 500 nm.

The model AGEs generated on MB from mel in dry (MWG) and aqueous conditions (ACG) were analyzed in terms of their absorption and fluorescence properties. The solution of the MB that was used for glycation displayed a characteristic maximum absorbance at 409 nm (Figure 2A, orange line), which was also shown by others [35]. The same maximum was shown by the MB-derived glycation products formed from mel (Figure 2A, grey solid and dashed line); however, the absorbance intensity at 409 nm was significantly decreased in the glycated proteins (0.7 AU and 1.3 AU for MWG and ACG products, respectively). This effect of decreased absorbance was especially prominent in the sample of MB-mel formed during MWG (Figure 2A, solid grey line) in comparison to the native MB solution (1.6 AU). The difference in absorbance intensity at 409 nm between MWG and ACG products confirms different structures of MB-mel formed in dry and aqueous conditions.

The fluorescence spectrum of AGEs is usually recorded upon excitation at 350 nm. At first, the unmodified MB protein was analyzed and exhibited a prominent emission at 397-400 nm in addition to a much less intense peak at 433–440 nm (Figure 2B, orange line). The same spectrum was observed for the protein modified with mel under aqueous conditions (MB-mel ACG; Figure 2B, grey dashed line). However, about a three-times increase in emission intensity (from 311 to 995 AU) was recorded at 397 nm for the MB-mel formed in dry conditions (MWG, Figure 2B, solid grey line). Similarly, an increase of about ten-times in fluorescence emission was observed for this AGE at 431 nm in comparison to the native MB protein or the MB-mel generated during conventional glycation (1615, 149, and 131 AU, respectively). There was also enhanced emission beyond 450 nm for MB-mel MWG, which was observed neither for native nor for the MB-mel formed during conventional glycation.

### 2.3. Fourier-Transform Infrared Spectroscopy (FTIR) of the Glycated Myoglobin

**Figure 3:**
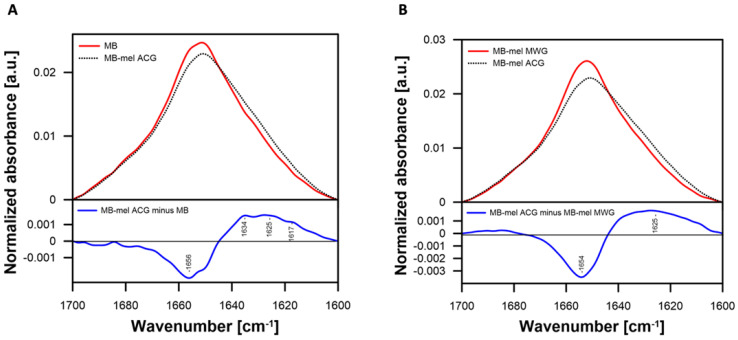
FTIR spectra were recorded in the amide I region. (**A**) A comparison of spectra recorded for Myoglobin (MB) and the glycated protein MB-mel ACG or (**B**) MB-mel MWG and MB-mel ACG is presented. The lower panels present the difference spectra calculated based on the spectra in the upper panels (as explained in the legends). The spectra in the upper panels are area-normalized.

The structural changes in proteins modified by melibiose were additionally confirmed using FTIR analysis. The spectra recorded in the amide I region of Myoglobin (MB) and the protein subjected to glycation in solution (MB-mel ACG) were compared with each other (Figure 3). The observed spectral differences are prominent for modification of the secondary structure and molecular organization of MB that can be assigned to glycation. The negative band in the difference spectrum (Figure 3A lower panel) centering at 1656 cm^−^^1^ and the positive bands, overlapping in the spectral region between 1617 and 1634 cm^−^^1^, can be interpreted in terms of a formation of parallel β-sheet and aggregated strand structures at the expense of α-helix. The fact that similar difference spectra were observed between the MB-mel ACG and MB-mel MWG (Figure 3B) indicates that similar protein reorganization takes place in both glycation conditions, although the extent of structural changes is more pronounced at room temperatures (compare the lower panels of Figure 3A and Figure 3B).

### 2.4. Analysis of the Site-Specific Modifications Present on MB Glycated with Mel

The amino acid residues modified in the MB-mel MWG and MB-mel ACG in comparison to the native MB protein were identified using LC-MS/MS analysis. The proteins were digested with the Lys-C enzyme (Promega, Madison, WI, USA) to generate peptides and allow for the identification of the sites modified during glycation. In this analysis, we performed the targeted screening for the known AGEs structures including CML, CEL, pyrraline, pentosidine, GAP, MG-H1, GOLD, MOLD, GA, GLX, and intact Hex/FL or mel). The native MB protein used in the experiments revealed seven peptides containing different AGE structures, presented in Table 1. The major modification found in the MB sample was CML identified at the lysine residues (K56, K63, K78, K87). There were also lysine residues modified by CEL (K62, K77, K145) and MGO (K56). We also found a peptide modified with one Hex identified at the histidine 64 (H64). In contrast, no arginine residues were found to be glycated.

Next, we analyzed peptides obtained after the digestion of MB-mel MWG with Lys-C. The resulting peptide list is presented in Table 2. Within the analyzed peptides, there were 34 fragments modified with different AGEs. MGO and its derivatives were the most abundant modifications identified on Lys (K56, K96, K98, K102, K145, K147), His (H93, H97), and Arg (MG-H1—R139). The second abundant modification was mel residue identified on Lys (K62, K63, K77), His (H64, H97), and Arg (R139), followed by Hex on Lys (K62, K63, K77, K96, K98) and His (H93). The MWG product also contained a CML structure, such as on the native MB protein, and an additional modification present on a few Lys residues (K77, K96, K98). Furthermore, several other modifications were found on the lysine residues, including MOLD (K63, K77, K78), GOLD (K78, K87), DOGDIC (K78, K79), GAP (K78), pyrraline (K96), GA (K98), and GLX (K96). Histidine residues were also modified by GA (H97, H113, H116) and GLX (H93, H119).

Remarkably, the number of AGE-modified peptides found in the MB-mel ACG product was smaller compared to the MWG product and comprised 21 peptides (Table 3). The most abundant modification in the ACG product was CML detected at Lys residues: K63, K77, K78, K79, and K145, some of which coincide with the modifications present in the native MB protein. Another abundant modification found was also the GA structure identified at Lys (K62, K63, K79, K87) and His (H81, H82). An intact mel modification was identified at Lys (K56, K63, K87) and His (H64, H81, H82) residues within AGE formed under aqueous conditions. Furthermore, lysine residues were also modified by: Hex (K56, K62, K77), CEL (K62, K77, K79, K145), MOLD (K47, K62, K63, K77), GOLD (K87, K133), DOGDIC (K78), pentosidine (K62), GLX (K77), and MGO (K56), while the histidine residues were modified by Hex (H64) and GLX (H64, H113).

**Figure 4:**
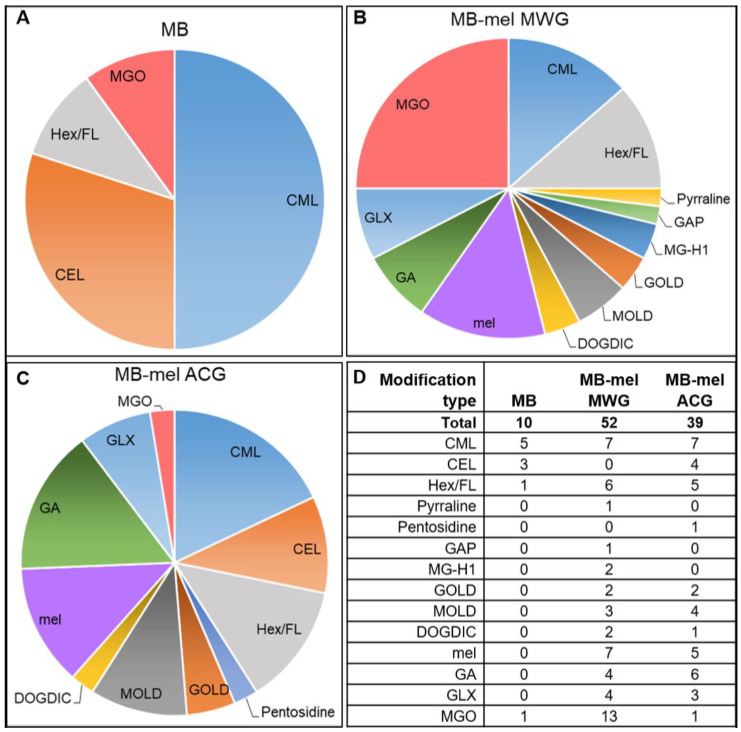
Comparison of the modification incidents (modified residues containing the indicated modification) identified in native MB protein (**A**) and MB-mel obtained in dry (MWG) (**B**) or aqueous glycation (ACG) (**C**) conditions. The number of identified modifications is presented in table (**D**).

The comparison of all modifications on the MB, MB-mel MWG, and MB-mel ACG displayed 10 different AGE structures on the native protein, 39 modifications on the ACG product, and 52 on the MB-mel formed under MWG conditions (Figure 4D). Initially, the MB protein presented CML (five residues) as the most prevalent AGEs (50% of all modifications), followed by CEL (three residues), MGO (1 residue), and Hex/FL (one residue) (Figure 4A). After glycation with mel, MGO/MG-H1 was the most abundant structure in the MWG product (found on 15 Lys, His, and Arg residues; Figure 3B). In contrast, only one MGO-modified amino acid (K56) was found in the ACG product and native MB (Figure 4A and C). This indicates a clear course of glycation by melibiose under anhydrous conditions towards the formation of AGE-MGO. The intact mel-AGE adduct (MAGE product) was found more frequently in the MWG than in ACG conditions (seven and five modifications, respectively, Figure 4D). This type of modification was not present in MB before glycation. The MWG and ACG conditions increased the number of CML (seven in both MWG and ACG products), Hex (six in MWG and five in ACG), and CEL (four modifications in ACG). Interestingly, the CEL structure was not found in the MB protein after MWG glycation. The GA formed more often under ACG than MWG conditions (six and four modifications, respectively), and the GOLD, MOLD, DOGDIC, and GLX structures occurred in similar amounts regardless of glycation conditions. Additionally, one pyrraline and one GAP modification were identified in the MWG product, while they were absent in the other samples. Pentosidine adduct was found only in the ACG product.

**Figure 5:**
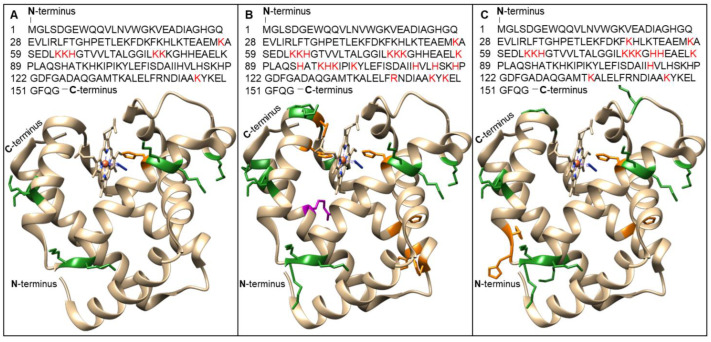
AGEs were identified on a MB protein before (**A**) and after modification with melibiose in dry conditions (**B**) and during aqueous conventional glycation (**C**). One letter sequence with amino acid residues modified with AGEs indicated in red in top panels and a three-dimensional structure model (created in Chimera 1.14 (RBVI)) of MB protein with the marked glycated sites (green—K, orange—H, pink—R residues) are presented in lower panels.

The identified modification sites on the MB and MB-mel products were visualized on a three-dimensional structure model of myoglobin and are shown in Figure 5. Initially, on the native MB, we identified eight amino acid residues carrying different AGE structures: seven Lys residues and one His residue (Figure 5A). In the MB-mel MWG product (Figure 5B), AGE adducts were detected at 19 amino acid residues: 12 Lys, six His, and one Arg. In the ACG product (Figure 5C), the total number of AGE-modified amino acids was 14, including 10 Lys and four His residues. Some of the AGE-modified His residues on the MB and both types of mel-derived AGEs are located at the center of interaction with the heme group—modified H64 in all samples and the H93 and H97 modified in the MWG product.

### 2.5. MAGE Adducts Consisting of an Intact Melibiose

Despite the identification of the intact mel moiety in a similar number of amino acid residues on the MB-mel MWG and ACG products (seven and five, respectively, incidents) (Figure 4 and Table 4), the dry conditions glycation generated an overall higher abundance of the intact mel AGE (21.9%) in comparison to the glycation in aqueous conditions (15.6%). In both cases, this intact mel moiety constituted 4.6 and 3.3% of all amino acids of the MB protein (Table 4).

**Figure 6:**
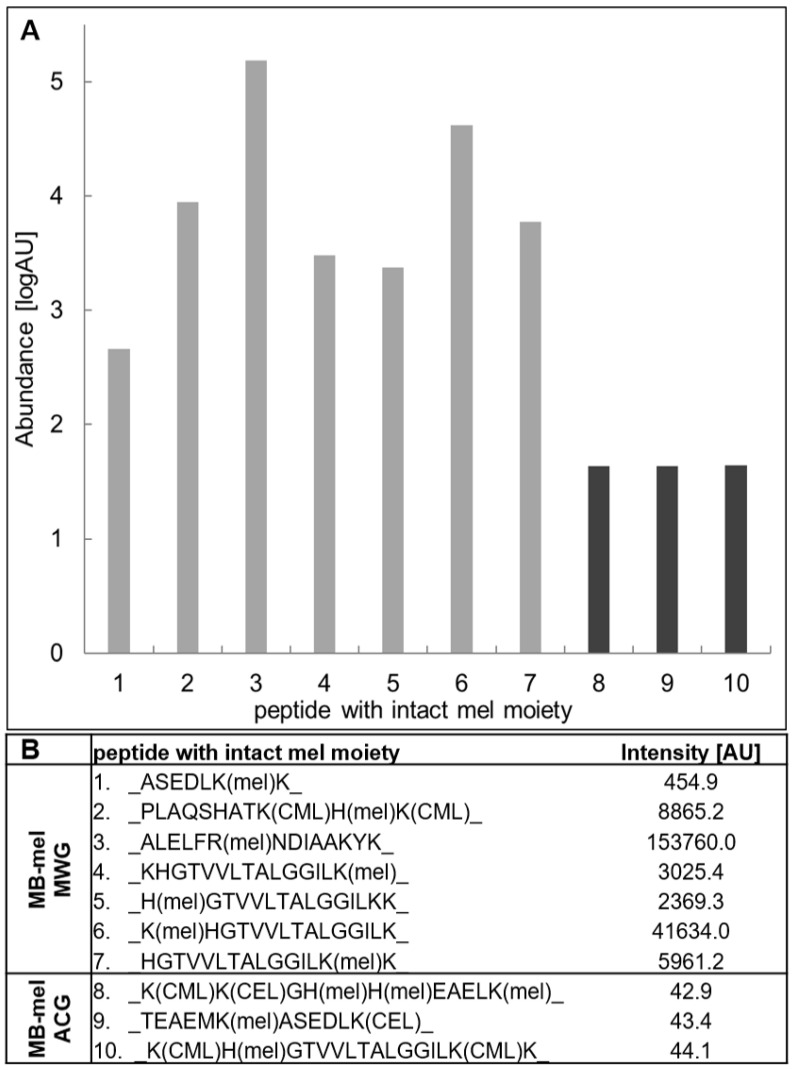
The abundance of the peptides modified with the intact mel moieties was identified on the glycated MB. Comparison of the abundance expressed as the log of MS signal intensities measured in arbitrary units (AU) recorded for peptides with the intact mel modification found in MB-mel MWG (light grey bars, peptides 1–7) and MB-mel ACG (dark grey bars, peptides 8–10) (**A**). The sequences of individual peptides (1–10) carrying the mel-modifications and their MS intensities are shown in table (**B**).

It is notifiable that the intensities of the peptides carrying caring the intact mel moiety shown in Figure 6B indicate the favored formation of this modification type in dry conditions. In MWG, the highest intensity of 153 760 AU was shown for the peptide comprising the mel-modified R (Figure 6A, peptide no. 3). The other peptides showed intensities ranging from 455 to 41 634 AU. There was substantially less mel bound to MB in the ACG product, with the peptide intensity between 42.9 and 44.1 AU (Figure 6A) in the three identified peptides (Figure 6B). Altogether, it suggests that aqueous conditions significantly reduce the efficiency of protein modification by mel and the formation of the moiety with intact mel.

## 3. Discussion

In many scientific studies on glycation, synthetic structural analogs of AGEs corresponding to the compounds present in vivo are used [36,37,38]. Model AGEs have been used to resolve the structure and properties of the formed compounds [39,40], to obtain specific antibodies [41,42,43], and to develop standards needed in clinical and diagnostic tests [26]. To understand the nature of the unconventional MAGE product found in various human and animal tissues [23,24], we characterized the extent of glycation and spatial amino acid modifications on the model protein. In our opinion, the glycation process carried out in aqueous conditions generates different antigenic structures that might be less relevant to the conditions in an organism. In this study, we used myoglobin, which often serves as a model for in vitro glycation with various sugars and aldehydes [44,45,46]. MB’s relatively low molecular mass (17 kDa) allows for the monitoring of the protein cross-linking. Moreover, MB having 19 lysyl, 11 histidyl, and two arginyl residues in the primary structure, is an easily glycated protein. So far, the most frequently used method to generate model AGEs is the conventional synthesis, carried out for several weeks in an aqueous medium or another polar solvent at a specific temperature [47]. Another method of AGEs generation under anhydrous conditions in a microwave reactor (for a significant reduction in reaction time from 21 days to 15-45 min) allows for the standardization of the reaction conditions and efficiency [23]; however, it generates AGEs with different antigenic properties [34].

In our study, MB glycated with mel analyzed by SDS-PAGE revealed a mixture of products with different molecular masses and degrees of cross-linking (Figure 1A). It remains in agreement with the literature on the formation of AGEs with different physicochemical properties from the same substrates, depending on the reaction conditions [34]. Our results indicate that MWG conditions favor the generation of products with a higher degree of cross-linking, more abundant mel load (Figure 1 and Figure 4, Table 4), and different antigenic properties (Figure 1B). The MAGE epitope is formed only under dry conditions, as we previously described [23]. FTIR analysis also revealed a noticeable change in the protein structure upon glycation under both conditions (Figure 3). However, the change was more pronounced in the MB-mel ACG that was generated in aqueous conditions. It suggests that under dry conditions, the protein structure is retained during the glycation reaction, which does not happen when the protein is solubilized in an aqueous buffer. We suggest that glycation under aqueous conditions promotes protein denaturation to a greater extent. The differences in physicochemical properties between the MWG and ACG glycation products were confirmed by absorption and fluorescence properties (Figure 2). The typical MB maximum absorption at 409 nm (corresponding to the heme molecule [35,48]) was lowered in AGEs formed from mel, and it is consistent with the literature [49,50]. This decrease in absorbance was almost two-times stronger in the MB-mel MWG product compared to the ACG one, suggesting the structural differences, as confirmed by FTIR analysis. The absorption spectrum of a heme group is, therefore, a direct indicator of its environment and, thus, the integrity of the protein structure, which is altered by glycation. A more profound decrease of 409 nm in the absorption of the MWG product compared to the ACG one can be explained with the modification of two instead of one His residues within a heme-binding site of an MB protein (as shown in Figure 5) and protein unfolding resulting from glycation load. Thus, the measurement of UV-Vis absorbance can be used to study the parameters of proteins, in particular their structure, and to obtain information on the binding and oxidation state of the heme prosthetic group. This method was initially used to measure glycated hemoglobin (HbA1c) in the blood of diabetic patients [51].

The structural difference between the ACG and MWG products became more obvious in the analysis of the fluorescence spectra. For AGEs, it is usually recorded in the emission range of 370–500 nm upon excitation at 350 nm [52]. The increase in the fluorescence emission of AGEs can be explained by the FRET analysis (Fluorescence Resonance Energy Transfer) [45]. While there was the same fluorescence spectrum with prominent emission at 397–400 nm for MB and MB-mel ACG product (Figure 2B), the MB-mel MWG presented the highest fluorescence intensity in the entire tested wavelength range. The maximum emission at 431 nm and enhanced emission beyond 450 nm, which is associated with the presence of AGEs [53], was unprecedented for the MB-mel MWG. It suggested that the presence of water during a glycation reaction significantly reduces the efficiency of MAGE formation observed as a reduced fluorescence at 431 nm. This might be explained by distinct water properties in the dry state of the protein in comparison to the solution. The water molecules, as an integral element on the protein surface, form a solvation layer even in the dried state after lyophilization, where there is no pure liquid water. The first solvation layer is an ordered structure made by the hydrogen bonds of water and held within the protein molecule, which facilitates the interaction with the ligand [54]. The studies by Merzel and Avbelj confirmed that a single pair of water molecules in the solvation layer could form stronger hydrogen bonds with each other if it is not surrounded by intercalating water molecules (such as in the water solution) and have a disturbing impact on the hydrogen bond network [55]. Moreover, the solvation layer on proteins under anhydrous glycation conditions mimics the cellular environment, with residual water molecules, rather than directly an aqueous solution.

The differences in the antigenic properties of MAGE were also revealed by the analysis of the spatial distribution within the protein of the known AGEs, using LC-MS/MS. Glycation reaction is mainly considered in the context of monosaccharides, and there are few reports on modification by disaccharides, mainly regarding food processing and lactose [56,57]. While melibiose is a disaccharide consisting of a galactose and glucose moiety linked by an α-1,6-glycosidic bond, Shinohar et al. proved that disaccharides linked by 1,6-glycosidic bonds are more active glycation factors than other disaccharides [58]. It is due to a higher rate of oxime-formation and acyclic formation compared to the disaccharides linked by 1-2, 1-3, and 1-4 bonds. In our study, depending on the method of glycation, the different panels of AGE adducts formed from mel were observed (Figure 4). Initially, in the native MB protein, there were already various AGE structures present, with CML being the most prevalent. It is the most often studied AGE and one of the most common in vivo AGE [59]. CML is produced by a series of transformations of the glucose–lysine condensation product, the successive rearrangement of the formed Amadori product, and subsequent oxidation, which ultimately yields CML. Another synthetic route involves the reaction between glyoxal and the amino group of lysine [60]. CEL, which was also found primarily on an MB protein, can be formed during a reaction of free amino groups with methylglyoxal [19]. The other modifications on MB, such as Hex (fructose or glucose), can be attached to the amino group of lysine, histidine, or arginine. The reaction between hexose and the ε-amino group of lysine creates fructoselysine (FL), which is an Amadori product and is further transformed into reactive dicarbonyls, precursors to other AGEs [19]. Raupbach et al. [49] also reported the presence of AGE structures in native MB, such as CML and CEL, but also MG-H1, pentosidine and pyrraline, which we did not confirm in our analysis. MB glycation under MWG or ACG conditions further increased the number of CML (seven in both MWG and ACG products) and CEL (four modifications in ACG) adducts. MGO/MG-H1 was the most abundant structure in the MWG, and only one MGO-modified amino acid was found in the ACG product and native MB. This clear course of glycation by melibiose under anhydrous conditions towards the formation of AGE-MGO might have biological consequences since the accumulation of MGO (highly reactive dicarbonyl) has been implicated in the pathogenesis of type 2 diabetes, vascular complications of diabetes, cardiovascular disease, cancer, and disorders of the central nervous system [61]. MGO is generated in vivo as a breakdown product of a triosephosphate intermediate in the glycolytic process and is also produced through lipid peroxidation. Moreover, it can be generated from unstable Schiff bases in Maillard reactions through reverse aldol condensation and oxidative decomposition (known as the Namiki pathway) and by the Wolff pathway, i.e., the metal-catalyzed autooxidation and dehydration of glucose [62]. MGO directly interacts with free amino groups in proteins, which leads to the development of AGEs, resulting in a high degree of physiological damage. MGO and MGO-derived AGEs can have an impact on organs and tissues, affecting their functions and structure. AGE-MGO also exert cellular effects via their interaction with specific AGE receptors (RAGE), which triggers an inflammatory response [63].

Another structural difference found by LC-MS analysis of the MB-mel was the presence of other AGEs. Among modifications observed more often under ACG than MWG conditions were GA, and GOLD, MOLD, DOGDIC, while GLX occurred in similar amounts regardless of glycation conditions. GA and GLX are highly reactive intermediates of the Maillard reaction and are precursors during the cross-linking of proteins. The generation of glycolaldehyde in vivo occurs during the degradation of carbohydrates but also by the myeloperoxidase system at sites of inflammation. On the other side, GLX represents the oxidative pendant to glycolaldehyde. It can be formed directly from the autoxidation of glucose [64]. GOLD, MOLD, and DOGDIC are the best-known structures that cross-link two amino acid side chains. These modifications are especially important in the aging process of proteins with a long half-life, i.e., collagen. GOLD and MOLD are the structures that cross-link two lysine residues during a reaction with glyoxal and methylglyoxal, respectively, and DOGDIC forms from 3-deoxyglucosone as the cross-link of lysine and arginine residues [5,19]. Modifications by these cross-linking AGEs are pathological in contrast to MAGE (formed in dry conditions), which is also formed in vivo.

Furthermore, a comparison of all modifications found in MB and MB-mel formed in MWG and ACG conditions displayed 10 different AGE structures on the native protein, 39 modifications on the ACG product, and 52 on MB-mel MWG (Figure 4). These modifications were located on the eight amino acid residues in native MB, 14 amino acids in MB-mel ACG, and 19 amino acid residues in MB-mel MWG (Figure 4). Some of the AGE-modified histidine residues in MB and both MB-mel products are at the center of interaction with the heme group, which explains the previously discussed decrease in absorbance at 409 nm. It is reasonable to suggest that other heme-containing proteins will also be impacted by MAGE modification via heme binding. Therefore, the in vivo present AGEs mimicked by protein glycation with mel warrants further studies on their biological properties and potential involvement in the pathogenesis of glycation-related diseases.

In conclusion, the presented results of a site-specific modification analysis of MB-mel products indicate the formation of a variety of AGE adducts and loads, depending on the synthesis conditions. We found the glycation reaction in an aqueous solution to be less efficient compared to the dry conditions, resulting in a lower degree of modification on the model protein. Since the reaction under the MWG conditions better reflects the conditions inside the tissues (scarce of the intercalating water molecules), it should be reconsidered whether it is appropriate to use the model AGEs prepared commonly in solution when studying the glycation effects in living organisms. Based on our results, we suggest that the course of glycation with melibiose under anhydrous conditions initiates the formation of the MAGE epitope, which is further transformed into AGE-MGO. MAGE adduct recognized by the anti-MAGE monoclonal antibodies is generated more efficiently under glycation in the dry state than in conventional glycation in solution. The complete epitope (containing intact carbohydrate moiety) forms on a model protein by coupling melibiose to lysine, arginine, and histidine. The anti-MAGE antibody exhibits specific and exclusive reactivity with MAGEs formed in a dry state (MWG), which confirms our previous results, showing that anti-MAGE monoclonal antibodies do not react with the ACG product and with other known AGEs synthesized from such precursors as, e.g., glucose, lactose, glyoxal, methylglyoxal under both MWG and ACG conditions [23]. Moreover, this antibody recognizes MAGEs independently of the carrier protein, e.g., cross-linked MWG products of BSA-mel and rabbit IgG-mel [24]. In the future, the use of isotopically labeled substrates will be necessary to resolve the pathway leading to MAGE in different conditions. The source and pathway of MAGE formation in vivo are still unknown; thus, the structural analysis of the model MAGE is the first step in understanding the mechanism behind the formation of the structural analog present in several tissues of the human body. Since our recent findings have shown specific MAGE-modified blood proteins [24], further studies on spacial modifications of immunoglobulin and serum albumin are of high relevance in cancer and diabetic conditions associated with protein modification by MAGE.

## 4. Materials and Methods

All chemicals were from Sigma-Aldrich (Saint Louis, MO, USA) unless otherwise stated. The myoglobin from equine skeletal muscle (MB) was used for glycation in the form as purchased.

### 4.1. Synthesis of Protein AGEs

The glycation products used in this study were generated as described earlier using either conventional glycation in solution (ACG) or reaction under dry conditions (MWG) [23]. Briefly, the MiliQ water solution mixtures of a model protein MB and melibiose (mel) in a 1:100 molar ratio (protein:carbohydrate) were prepared, followed by the ACG or MWG reaction. In the ACG method, the MB/mel solution was incubated for 21 days at room temperature, while in the MWG method, an analogous MB/mel solution was lyophilized and incubated in a microwave initiator reactor (Biotage, Uppsala, Sweden) for 45 min at 85 °C. Next, the samples were dissolved in MilliQ water and centrifuged at 5000× *g* for 15 min to remove any insoluble precipitates. The obtained supernatants were dialyzed against MilliQ water on Amicon Ultra-15 centrifuge filters (Merck Millipore, Burlington, MA, USA) with a 30 kDa cut-off to separate unreacted substrates and MB monomers. The resulting glycation products were lyophilized and stored at −20 °C for further characterization.

### 4.2. SDS-PAGE and Western Blotting

The MB and MB-mel products after were diluted 1:1 in 2× concentrated Laemmli buffer (Bio-Rad, Hercules, CA, USA) supplemented with 100 mM β-mercaptoethanol and denatured by incubation at 100 °C for 5 min. Then, the samples were loaded (10 µg/well) onto a polyacrylamide gel consisting of a 4% stacking and a 12% separation gel. The electrophoretic separation was carried out at a constant voltage of 100 V for 90 min in a Mini-Protean Tetra Cell system (Bio-Rad, Hercules, CA, USA). Next, the gel was stained with Coomassie Brilliant Blue solution (3 mM Coomassie blue, (*v*/*v*) 40% methanol, 10% acetic acid) or used for protein transfer.

For Western blotting analysis, the proteins separated on the polyacrylamide gel were transferred to a PVDF membrane (0.45 µm, Immobilon-P Transfer Membrane PVDF, Merck-Millipore, Burlington, MA, USA) using a Mini Trans-Blot Cell (Bio-Rad, Hercules, CA, USA). The transfer was carried out for 1 h at a constant voltage of 100 V. Then, the membrane was blocked in a solution of 5% powdered skimmed milk (SM Gostyń, Poland) in TBS-T (20 mM Tris-HCL, 150 mM NaCl, pH 7.4 with 0.05% Tween-20) at room temperature for 1 h. After washing 3 times for 5 min in TBS-T, the membrane was incubated overnight at 4 °C with a culture medium collected from the hybridoma cells containing mouse anti-MAGE monoclonal antibodies [23]. Then, excess antibodies were washed out 3 times with TBS-T solution, followed by incubation for 1.5 h at room temp with goat anti-mouse IgE-HRP secondary antibody (Origene, Rockville, MD, USA) diluted 1:5000 in PBS. The membrane was next washed 5 times with TBS-T and developed with a chemiluminescent solution (SuperSignal™ West Pico PLUS Chemiluminescent Substrate, Thermo Scientific, Waltham, MA, USA). The results were recorded using a PXi Touch detection and analysis system (SYNGENE, Cambridge, UK).

### 4.3. Absorption Spectra

The UV-Vis absorption spectra of 0.5 mg/mL aqueous solutions of MB-mel and native MB were collected within the 240–700 nm wavelength range on a 96-well quartz plate, using Epoch Microplate Spectrophotometer (BioTek Instruments, Winooski, VT, USA).

### 4.4. Fluorescence Spectra

The fluorescence spectra in a range of 370–500 nm were obtained using the EnSpire Multimode Plate Reader spectrofluorometer (PerkinElmer, Waltham, MA, USA) with excitation at a wavelength of 350 nm. The measurements were carried out on 0.5 mg/mL aqueous solutions of 100 µL/well MB-mel and native MB using a black 96-well plate.

### 4.5. Fourier-Transform Infrared Spectroscopy (FTIR)

The lyophilized protein samples were dissolved in a H_2_O:D_2_O mixture (95:5, by volume), deposited to an ATR crystal by evaporation under a stream of argon and subjected to FTIR analysis. A mixture of H_2_O and D_2_O was applied to correct spectra for possible remaining bulk water in samples after deposition. IR absorption spectra were recorded with a Nicolet iS50 FTIR spectrometer from Thermo Scientific (USA) equipped with a GladiATR monolithic diamond crystal from Pike Scientific (USA), with a single reflection attenuated total reflection setup. Typically, 10 scans were collected, Fourier-transformed, and averaged for each measurement. The absorption spectra at a resolution of one data point every 4 cm^−1^ were obtained in the region between 4000 and 400 cm^−1^. An increase in the absorption spectra was monitored in the amide I region. Spectral analysis was performed using the GRAMS/AI v9.0 software from Thermo Scientific (USA).

### 4.6. Mass Spectrometry Analysis

The samples for LC-MS/MS analysis were prepared using the Filter-Aided Sample Preparation (FASP) method [65]. Briefly, the samples of MB-mel MWG, MB-mel ACG, and native MB (80 µg) were diluted 1:1 in a sample buffer (100 mM Tris-HCl pH 8.0, 50 mM DTT (dithiothreitol) containing 1.5% SDS (sodium dodecyl sulfate)) and were incubated for 10 min at 96 °C. Then, 250 µL of UAD buffer (8 M urea, 100 mM Tris-HCl, pH 8.5, 1 mM DTT) was added to each sample before application onto a tube filter (Microcon, Merck Millipore, Burlington, MA, USA) with a 30 kDa cut-off. Next, the tubes were centrifuged for 25 min at 10,000× *g*. The filters were next washed 6 times with UAD buffer (250 µL) and centrifuged each time for 20 min at 10,000× *g*. Subsequently, the samples were washed 3 times with DBD buffer (50 mM Tris-HCl, pH 8.5, 1 mM DTT) and centrifuged each time for 20 min at 10,000× *g*. Next, 80 µL of Lys-C enzyme (Promega, Madison, WI, USA) solution in DBD buffer (2 µg/80 µL) was added to each sample. Enzyme digestion was carried out overnight at 37 °C. The next day, the samples were centrifuged for 10 min at 10,000× *g* and further washed 2 times with DBD buffer (100 and 125 µL), centrifuged for 10 min at 10,000× *g*, collecting the eluate after each centrifugation step, and pooling into one tube. Then, the concentration of the obtained peptides was determined by measuring tryptophan fluorescence (WF test), as described previously [66]. From each sample, 8 µg of the peptide mixture was desalted on the C18 membrane (C18 Empore Disk, 3M, Saint Paul, MN, USA), according to the StageTips (stop and go extraction tips) method [67]. Finally, the samples were concentrated for 30–40 min under vacuum to the final volume of 10 µL, using Concentrator Plus (Eppendorf, Hamburg, Germany) set up at 45 °C. The obtained samples of peptide mixtures were analyzed using the LC-MS/MS system (LTQ Orbitrap, Thermo Scientific, Waltham, MA, USA) according to the previously described method [68].

### 4.7. Proteomic Data Analysis

The MS data were analyzed using MaxQuant v.1.6.10.43 software with the Andromeda search engine to identify and quantify glycation-associated modifications. The monoisotopic masses corresponding to the known AGE structures and potential glycating substrates present in the human body were introduced into the software (Table 5) based on the UNIMOD database (www.unimod.org, accessed on 10 February 2020) or the structural formula of the considered AGE structures.

## Figures and Tables

**Table 1:** Glycation adducts identified in native MB.

Type of Modification	Modified Residue	Detected m/z	∆m [Da]	Score	Peptide Sequence
**CML**	K78	412.225	+58.005	6.89 × 10^14^	_K(CML)KGHHEAELK_
**CML**	K87	412.224	+58.005	3.76 × 10^14^	_KKGHHEAELK(CML)_
**CML**	K56	470.553	+58.005	5.69 × 10^14^	_TEAEMK(CML)ASEDLK_
**CEL**	K145	716.894	+72.021	6.9 × 10^14^	_ALELFRNDIAAK(CEL)_
**CML**	K63	522.318	+58.005	1.97 × 10^14^	_K(CML)HGTVVLTALGGILK_
**Hex, CML 2× CEL**	H64, K63, K62, K77	1257.686	+364.100	5.35 × 10^14^	_ASEDLK(CEL)K(CML)H(Hex)GTVVLTALGGILK(CEL)_
**MGO**	K56	469.222	+54.010	4.69 × 10^14^	_TEAEMK(MGO)ASEDLK_

CML—carboxymethyllysine; CEL—carboxyethyllysine; Hex—hexose/fructoselysine; MGO—methylglyoxal.

**Table 2:** Glycation adducts identified in MB-mel formed during glycation in dry conditions (MWG).

Type of Modification	Modified Residue	Detected m/z	∆m [Da]	Score	Peptide Sequence
**2× Hex**	K63, K77	611.018	+324.106	7.45 × 10^14^	_K(Hex)HGTVVLTALGGILK(Hex)_
**3× MOLD**	K63, K77, K78	594.690	+147.024	3.97 × 10^14^	_K(MOLD)HGTVVLTALGGILK(MOLD)K(MOLD)_
**CML**	K87	412.224	+58.005	1.75 × 10^14^	_KKGHHEAELK(CML)_
**CML**	K96	425.898	+58.005	3.81 × 10^14^	_PLAQSHATK(CML)HK_
**CML**	K56	470.553	+58.005	8.26 × 10^14^	_TEAEMK(CML)ASEDLK_
**CML**	K77	782.974	+58.005	9.56 × 10^14^	_HGTVVLTALGGILK(CML)K_
**CML**	K63	522.319	+58.005	7.59 × 10^14^	_K(CML)HGTVVLTALGGILK_
**GAP**	K78	539.325	+109.029	7.48 × 10^14^	_HGTVVLTALGGILKK(GAP)_
**GOLD, DOGDIC**	K78, K79	668.834	+160.016	5.56 × 10^14^	_K(GOLD)K(DOGDIC)GHHEAELK_
**GOLD, DOGDIC**	K87, K78	668.834	+160.016	4.61 × 10^14^	_K(DOGDIC)KGHHEAELK(GOLD)_
**Hex**	K62	476.747	+162.053	5.48 × 10^14^	_ASEDLK(Hex)K_
**Hex**	H93	611.018	+162.053	4.7 × 10^14^	_PLAQSH(Hex)ATKHKIPIK_
**Hex**	K98	611.017	+162.053	4.31 × 10^14^	_PLAQSHATKHK(Hex)IPIK_
**Hex**	K96	611.018	+162.053	2.39 × 10^14^	_PLAQSHATK(Hex)HKIPIK_
**mel**	R139	659.345	+324.106	4.39 × 10^14^	_ALELFR(mel)NDIAAKYK_
**mel**	K62	557.771	+324.106	3.57 × 10^13^	_ASEDLK(mel)K_
**mel**	K63	611.018	+324.106	4.88 × 10^14^	_K(mel)HGTVVLTALGGILK_
**mel**	H64	611.018	+324.106	6.83 × 10^14^	_H(mel)GTVVLTALGGILKK_
**mel**	K77	611.018	+324.106	4.32 × 10^14^	_KHGTVVLTALGGILK(mel)_
**mel**	K77	611.019	+324.106	7.53 × 10^14^	_HGTVVLTALGGILK(mel)K_
**mel, 2× CML**	H97, K96, K98	553.268	+440.116	7.05 × 10^14^	_PLAQSHATK(CML)H(mel)K(CML)_
**MG-H1**	R139	569.313	+54.010	4.32 × 10^14^	_ALELFR(MG-H1)NDIAAKYK_
**MG-H1**	R139	472.261	+54.010	8.01 × 10^14^	_ALELFR(MG-H1)NDIAAK_
**Pyrraline**	K96	593.008	+108.021	1.18 × 10^14^	_PLAQSHATK(Pyrraline)HKIPIK_
**4× GA**	H97, K98, H113, H116	1003.870	+408.128	2.72 × 10^14^	_H(GA)K(GA)IPIKYLEFISDAIIH(GA)VLH(GA)SK_
**GLX**	K96	496.762	+39.995	1.18 × 10^14^	_PLAQSHATK(GLX)_
**GLX**	H119	771.837	+39.995	3.32 × 10^14^	_H(GLX)PGDFGADAQGAMTK_
**2× GLX**	H93, K96	516.761	+79.99	3.93 × 10^14^	_PLAQSH(GLX)ATK(GLX)_
**MGO**	K56	469.224	+54.010	4.34 × 10^14^	_TEAEMK(MGO)ASEDLK_
**MGO**	K147	569.313	+54.010	3.15 × 10^14^	_ALELFRNDIAAKYK(MGO)_
**2× MGO**	K96, K102	593.008	+108.020	1.18 × 10^14^	_PLAQSHATK(MGO)HKIPIK(MGO)_
**5× MGO**	H93, K96, H97, K98, K102	647.021	+270.050	1.15 × 10^14^	_PLAQSH(MGO)ATK(MGO)H(MGO)K(MGO)IPIK(MGO)_
**3× MGO**	H93, K98, K102	611.010	+162.030	3.62 × 10^14^	_PLAQSH(MGO)ATKHK(MGO)IPIK(MGO)_
**MGO**	K145	472.261	+54.010	5.68 × 10^14^	_ALELFRNDIAAK(MGO)_

CML—carboxymethyllysine; Hex—hexose/fructoselysine; GAP –glycolaldehyde pyridine; MG-H1—methylglyoxal 5-hydro-5-methylimidazolone; GOLD—glyoxal-lysine dimer; MOLD—methylglyoxal-lysine dimer; DOGDIC—3-deoxyglucosone-derived imidazolium cross-link; mel—melibiose; GA—glycolaldehyde; GLX—glyoxal; MGO—methylglyoxal.

**Table 3:** Glycation adducts identified in MB-mel formed in aqueous conventional glycation (ACG).

Type of Modification	Modified Residue	Detected m/z	∆m [Da]	Score	Peptide Sequence
**2× Hex**	K56, K62	559.253	+324.106	8.10428 × 10^14^	_TEAEMK(Hex)ASEDLK(Hex)_
**2× Hex, 2× CML**	H64, K77, K78, K79	692.390	+324.106	7.97853 × 10^14^	_H(Hex)GTVVLTALGGILK(Hex)K(CML)K(CML)_
**3× mel,** **CML, CEL**	H81, H82, K87, K78, K79	760.337	+1102.344	6.10181 × 10^14^	_K(CML)K(CEL)GH(mel)H(mel)EAELK(mel)_
**3× MOLD**	K62, K63, K77	766.430	+147.024	7.30365 × 10^14^	_ASEDLK(MOLD)K(MOLD)HGTVVLTALGGILK(MOLD)_
**CEL**	K145	478.264	+72.021	8.29719 × 10^14^	_ALELFRNDIAAK(CEL)_
**CEL**	K77	725.934	+72.021	3.53221 × 10^14^	_HGTVVLTALGGILK(CEL)_
**CML**	K77	782.973	+58.005	3.3215 × 10^14^	_HGTVVLTALGGILK(CML)K_
**CML**	K145	570.644	+58.005	6.11103 × 10^14^	_ALELFRNDIAAK(CML)YK_
**GOLD**	K133	769.332	+34.992	4.66304 × 10^14^	_HPGDFGADAQGAMTK(GOLD)_
**GOLD, DOGDIC**	K87, K78	668.834	+160.016	8.9105 × 10^14^	_K(DOGDIC)KGHHEAELK(GOLD)_
**Hex**	K62	476.746	+162.053	1.08165 × 10^14^	_ASEDLK(Hex)K_
**mel, 2× CML**	H64, K63, K77	692.390	+440.116	6.8908 × 10^14^	_K(CML)H(mel)GTVVLTALGGILK(CML)K_
**mel, CEL**	K56, K62	583.261	+396.127	5.48394 × 10^14^	_TEAEMK(mel)ASEDLK(CEL)_
**MOLD**	K47	556.313	+49.008	4.13698 × 10^14^	_FDKFK(MOLD)HLK_
**Pentosidine**	K62	737.084	+58.992	5.06918 × 10^14^	_ASEDLK(Pentosidine)KHGTVVLTALGGILK_
**2× GA**	K62, K63	332.171	+204.064	2.58266 × 10^14^	_ASEDLK(GA)K(GA)_
**4× GA**	K79, H81, H82, K87	486.233	+408.128	9.06642 × 10^14^	_K(GA)GH(GA)H(GA)EAELK(GA)_
**GLX**	H64	709.921	+39.995	3.29039 × 10^14^	_H(GLX)GTVVLTALGGILK_
**GLX**	K77	473.617	+39.995	8.98953 × 10^14^	_HGTVVLTALGGILK(GLX)_
**GLX**	H113	642.341	+39.995	8.29994 × 10^14^	_YLEFISDAIIH(GLX)VLHSK_
**MGO**	K56	469.223	+54.010	3.19781 × 10^14^	_TEAEMK(MGO)ASEDLK_

CML—carboxymethyllysine; CEL—carboxyethyllysine; Hex—hexose/fructoselysine; GOLD—glyoxal-lysine dimer; MOLD—methylglyoxal-lysine dimer; DOGDIC—3-deoxyglucosone-derived imidazolium cross-link; mel—melibiose; GA—glycolaldehyde; GLX—glyoxal; MGO—methylglyoxal.

**Table 4:** Summary of the nucleophilic amino acid residues modified with intact mel.

Glycation Type	Residue (Amount within MB)	Residues with Intact Mel	% of Residue Type	% of Nucleophilic Residues (32 aa)	% of Total Residues (153 aa)
**MWG**	K (19)	4	21.1	21.9	4.6
	H (11)	2	18.2		
	R (2)	1	50.0		
**ACG**	K (19)	2	10.5	15.6	3.3
	H (11)	3	27.3		
	R (2)	0	0.0		

MB—myoglobin; mel—melibiose; MWG—microwave glycation in a dry state; ACG—conventional glycation in solution.

**Table 5:** AGE modifications considered in the analysis and the corresponding monoisotopic masses.

AGE Modification Type	Monoisotopic Mass [Da] [Da] [Da]
**CML**	58.005
**CEL**	72.021
**Hex/FL**	162.053
**Pyrraline**	108.021
**Pentosidine**	58.992
**GAP**	109.029
**MG-H1 (MGO on R)**	54.010
**GOLD**	34.992
**MOLD**	49.008
**DOGDIC**	125.024
**mel**	324.106
**GA**	102.032
**GLX**	39.995
**MGO (on K, H)**	54.010

CML—carboxymethyllysine; CEL—carboxyethyllysine; Hex/FL—hexose/fructoselysine; GAP –glycolaldehyde pyridine; MG-H1—methylglyoxal 5-hydro-5-methylimidazolone; GOLD—glyoxal-lysine dimer; MOLD—methylglyoxal-lysine dimer; DOGDIC—3-deoxyglucosone-derived imidazolium cross-link; mel—melibiose; GA—glycolaldehyde; GLX—glyoxal; MGO—methylglyoxal; R—arginine; K—lysine; H—histidine.

## Data Availability

The raw data from mass spectrometry analysis can be shared upon request.
